# Social media as a platform for science and health engagement: challenges and opportunities

**DOI:** 10.1186/s13584-016-0114-3

**Published:** 2016-11-21

**Authors:** Graham Dixon

**Affiliations:** The Edward R. Murrow College of Communication, Washington State University, Murrow Hall 106a, Pullman, WA 99163 USA

## Abstract

Social media has become a major platform for debates on science and health. This commentary argues that while social media can present challenges to communicating important health matters, it can also provide health experts a unique opportunity to engage with and build trust among members of the public.

## Background

Covering the 2013 polio vaccination crisis in Israel, Daniela Orr and colleagues investigate the divergent roles that news media and social media play in public engagement of scientific debates. In doing so, they provide a comprehensive content analysis that speaks to the challenges and opportunities health officials face when communicating science in a constantly changing media landscape. While focused on the polio crisis in Israel, their article illustrates broad themes that can be applied to other scientific debates in the world. Importantly, their findings shed light on how social media can be used for evidence-based science engagement with skeptical audiences.

The article first explores the differences in how news media sources and social media engaged with the crisis. For example, Orr and her colleagues find mainstream Israeli press outlets primarily used government health agencies as sources without covering alternative views, such as those within the anti-vaccine communities. This finding stood in stark contrast to the coverage within Facebook groups dedicated to the polio crisis, which provided outlets for communities not covered by news media to express health-related concerns. Indeed, news media has become aware that providing space to opposing views unsubstantiated by scientific evidence can be problematic [1]. Studies have documented that “false balance” of vaccine debates can lead to misperceptions about vaccine safety and its non-existent link to autism [2, 3]. Thus, while mainstream news outlets eschew unscientific claims as a measure of ensuring accuracy and ethical standards, social media provides a platform for alternative views to flourish.

### Social media: challenges and opportunities

The emergence of social media has presented numerous challenges in how health organizations respond to and engage with public health controversies. With layers of bureaucratic rules, limited social media training, and a lack of continuous social media monitoring, many health organizations – particularly those in the public sector – often struggle to swiftly and effectively respond to social media postings. These challenges are further compounded by social media postings dedicated to alternative health views that attack practices vital to maintaining public health, such as vaccination. Indeed, websites dedicated to anti-vaccine views can have significant negative effects on people’s vaccine risk perceptions and intentions. In one study, participants who viewed vaccine-critical websites for 5 to 10 min were more likely to believe vaccinating was risky and reported decreased intentions to vaccinate [4]. As a result, social media pages that encourage the exchange and promotion of anti-vaccine information are often viewed by health experts as threats to health promotion and disease prevention. However, Orr and her colleagues suggest that social media is not wholly dedicated to anti-vaccine tropes and echo-chambers. Instead, their findings suggest social media can be an opportunity for more effective public engagement in science.

Orr and her colleagues show that among anti-vaccine Facebook groups highlighted in their study, distrust and skepticism in the Israeli Ministry of Health emerged as a key theme in public posts. On the other hand, the more impartial *Parents Talk about the Polio Vaccination* Facebook group appears to bridge the divide between hyper-partisan anti-vaccine groups with the one-sided news media. In particular, this Facebook group acted as a platform that joined concerned parents and anti-vaccine activists with medical experts who employed an evidence-based medicine approach toward engagement. Though this engagement led to public debates on vaccine safety and conflicting information, the type of engagement conducted by medical experts via social media could ameliorate the negative effects of conflicting vaccine information. In fact, recent research suggests that scientific evidence can be accurately conveyed to the public even when opposing views are presented. One method for reducing the effects of false balance is to provide meaningful context on which side of a two-sided argument is supported by experts and evidence. However, studies by colleagues and I caution that simply including this type of information does little to sway those whose views already run counter to the scientific consensus [3, 5]. Instead, engendering greater trust in physicians and health organizations can be an influential factor for persuading initially skeptical audiences to commit to treatment recommendations [6]. Though anecdotal, Orr and her colleagues illustrate this point when describing a multi-participant discussion post from a mother concerned about the polio vaccine’s health risks. In the post, the mother commented on how after consulting with a fellow member from the Facebook group she made her decision to vaccinate her child. The fellow member provided the mother medical documentation that convinced her to vaccinate her child. The interpersonal connection she made with the fellow Facebook member could have elicited greater trust in the scientific information she had received.

Additionally, the participation of healthcare professionals in the Facebook group also gave people a platform to engage with expert sources – something that is not readily available in mainstream news media organizations. Physician engagement in social media could help repair trust in medical expertise and organizations among anti-vaccine communities. Though the authors’ content analysis cannot provide evidence on whether this occurred, it is a reasonable possibility to explore with additional studies. Therefore, future research must consider whether the types of social media engagement documented in the article result in measurable improvements to institutional trust, as well as health promotion behavior and beliefs.

## Conclusion

In conclusion, social media is often viewed as a barrier to effective science and health communication due to it being a platform for the free enterprise of ideas, some of which run counter to scientific/medical evidence, and as a result, pose threats to community health. Indeed, conflicting scientific information for issues of great importance, like polio vaccination, can lead to greater uncertainty around established science. However, unlike mainstream media coverage, social media allows for opportunities in science engagement that rarely exist through other media platforms. As noted in the article, this engagement could improve trust in medical institutions, government agencies, and experts– a factor that is an important driver for medical acceptance. Thus, while allowing for a greater diversity of viewpoints, social media could be a key platform used by government health agencies and medical experts for addressing the core factors behind skepticism in modern medicine.
